# Oncoplastic breast surgery combining partial mastectomy with resection of double equilateral triangular skin flaps

**DOI:** 10.1007/s00595-021-02355-w

**Published:** 2021-08-16

**Authors:** Yuko Kijima, Munetsugu Hirata, Naotomo Higo, Hiroko Toda, Yoshiaki Shinden

**Affiliations:** 1 Department of Breast Surgery, School of Medicine, Fujita Health University, 1-98 Dengakugakubo, Kutsukake-cho, Toyoake, Aichi 470-1192 Japan; 2 Department of Digestive Surgery, Breast and Thyroid Surgery, Kagoshima University Graduate School of Medical and Dental Sciences, 8-35-1 Sakuragaoka, Kagoshima, 890-8520 Japan

**Keywords:** Breast cancer, Oncoplastic breast surgery, Double triangular skin resection

## Abstract

The treatment of early breast cancer using oncoplastic breast surgery (OBS) has been gradually increasing in popularity and is recognized for its efficacy in local control and excellent cosmetic results. We herein report a useful technique for obtaining symmetry of the breast shape for an early breast lesion located in an outer area, close to the nipple-areola, in a Japanese patient with ptotic, fatty breasts. We designed two equilateral triangles: one just upon the resected area and the other on the axilla. They were located on a straight line, with one top pointed to the cranial side and one to the caudal side. A crescent area around the areola was de-epithelialized in the 12 o’clock and 6 o’clock directions. Columnar-shaped breast tissue and an equilateral triangular skin flap and fatty tissue were removed together. To fill the defect, a skin-glandular flap was slid horizontally after suturing the inframammary line. Although an incision scar was formed on the breast and lateral chest wall in a Z-shape, this new technique was able to achieve not only cancer control but also excellent cosmetic results.

## Introduction

Breast conservation therapy (BCT) is well established as a treatment for breast cancer that provides local disease control with acceptable cosmetic results [1]. However, the possibility of insufficient resection margins may increase the risk of local recurrence if too much attention is paid to cosmesis.

Oncoplastic breast surgery (OBS) is well known to achieve both cancer control and cosmetic satisfaction by removing sufficient tissue with the introduction of a plastic procedure.

We herein report a new technique that was useful for managing a lesion where it was necessary to remove skin together with the lesion in the outer lower quadrant area of a ptotic breast.

### Patient

A 63-year-old Japanese patient diagnosed with early breast cancer was enrolled in this study. The indications for OBS combining partial mastectomy with resection of double equilateral triangular skin flaps were as follows: (1) the cancer lesion was restricted to the lateral area and close to the nipple-areola area; (2) informed consent was obtained preoperatively after an explanation of the surgical procedure; and (3) she was not very worried about the length of the operation scar. A digital camera with a resolution of 14.1 megapixels was used, and a blue panel was used as the background.

She was not receiving systemic chemo- or endocrine therapy for breast cancer diagnosed as cT1N0M0 Stage IA according to the TNM classification. The patient was seen by the breast surgeon (Y.K.) at an outpatient clinic and on the day before surgery. She received an explanation of the plan for the operation and other surgical options, e.g. other oncoplastic surgical techniques, such as J-mammoplasty [1].

### Design

**Fig. 1:**
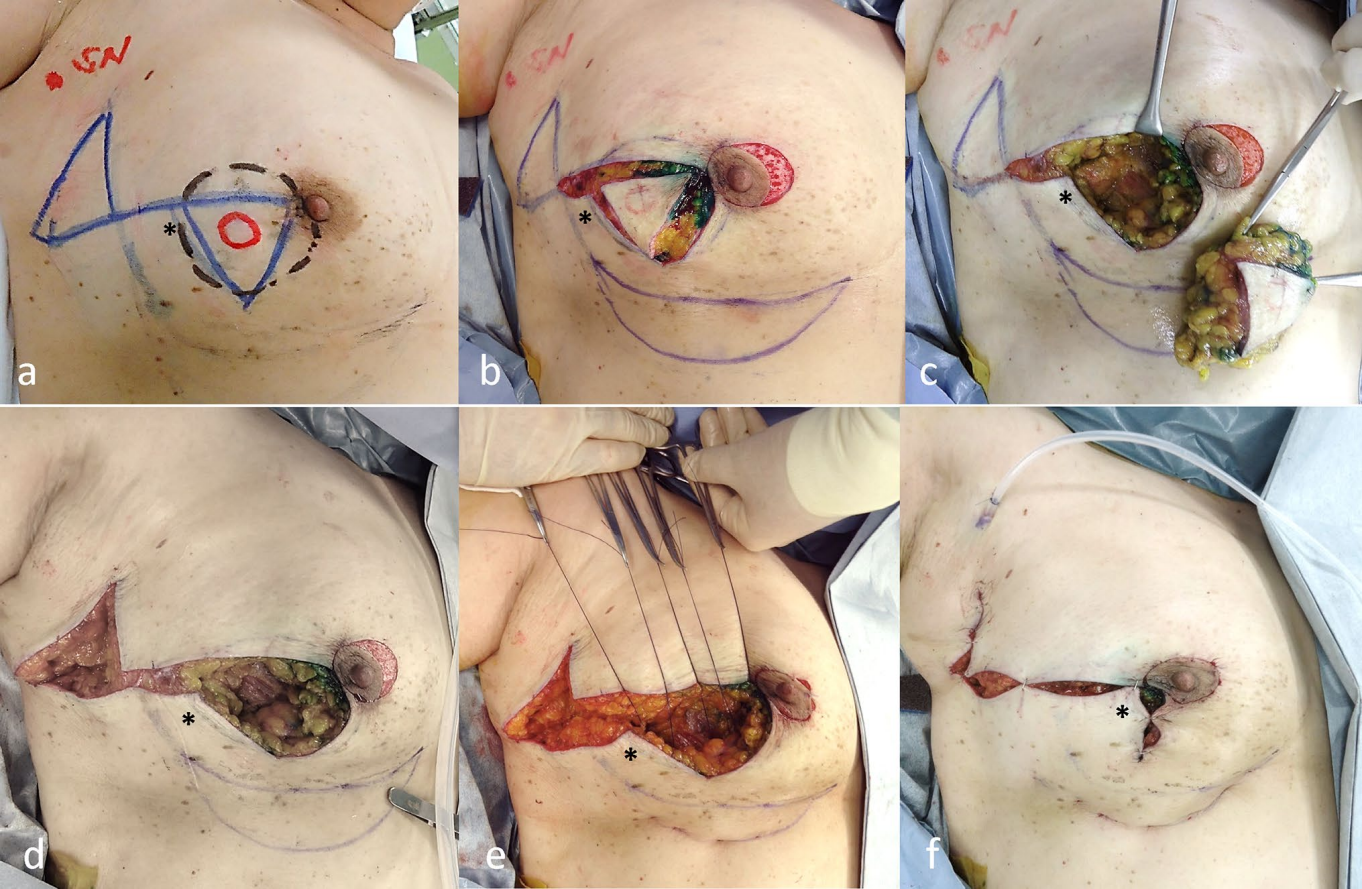
Oncoplastic breast surgery, partial mastectomy with resection of double equilateral triangular skin flaps. **a** Preoperative markings. Red circle: cancerous area; dotted pale black ink: resected area of the breast; blue ink (triangle) on the breast: resected skin; SN: sentinel lymph node. **b, c** Partial mastectomy was performed. Several sections were examined intraoperatively and diagnosed as being cancer-free. d. Another triangle of skin and fatty tissue was removed. The double triangular area was connected horizontally. e. A new inframammary line was created 2.0 cm below the true inframammary one. We laid down 2–0 PDS^®^ sutures in the subdermal layer and elevated them toward the cranial side. We tied them without fixing to the chest wall. f. The cranial and caudal skin-glandular flaps were horizontally slid to fill in the defects. *: Adjacent breast tissue and skin used to repair the defect

Partial mastectomy was planned with cylinder-shaped resection and 2.0-cm surgical margins. We also planned to remove two equilateral triangular skin flaps: one upon the resected area and the other in the lateral thoracic area (Fig. 1a). The two equilateral triangles were located on a straight line, with one top turned to the cranial side and the other to the caudal side.

### A sentinel lymph node biopsy

In all patients where a sentinel lymph node biopsy using the RI and dye method was performed via the same incision in the axillary area, the sentinel lymph nodes were biopsied and examined histologically during surgery. No sentinel lymph nodes were positive for metastasis in the present patient, so axillary lymphadenectomy was avoided.

### OBS

First, a crescent-shaped area located at the 12 o’clock to 6 o’clock directions along with the areola was de-epithelialized (Fig. 1b). Partial mastectomy with an equilateral triangular skin flap was then performed (Fig. 1c). The resected tissue weighed 68.3 g. Several sections were examined intraoperatively and diagnosed as being cancer free. An equilateral triangular skin flap with subdermal fatty tissue on the lateral-axillar area was then removed (Fig. 1d). A new inframammary line was designed on an area 2.0 cm lower than the true inframammary one. We laid down 2–0 PDS^®^ sutures in the subdermal layer and elevated them toward the cranial side; we then tied them without fixing to the chest wall. The sutured points were elevated toward the cranial side, resulting in the new inframammary line being clear (Fig. 1e).

To fulfill the inner defect, a skin-glandular flap was slid horizontally from the lateral area to the inner defect (Fig. 1f). A corner of the lower skin-glandular flap was moved to match the medial-upper edge, and the upper one of the cranial skin-glandular one was moved to the lateral-lower one (Fig. 1e). A closed suction drainage line was placed onto the surface of the major pectoral muscle. Several points were sutured so that the sutured sections formed a Z-shape (Fig. 1f).

**Fig. 2:**
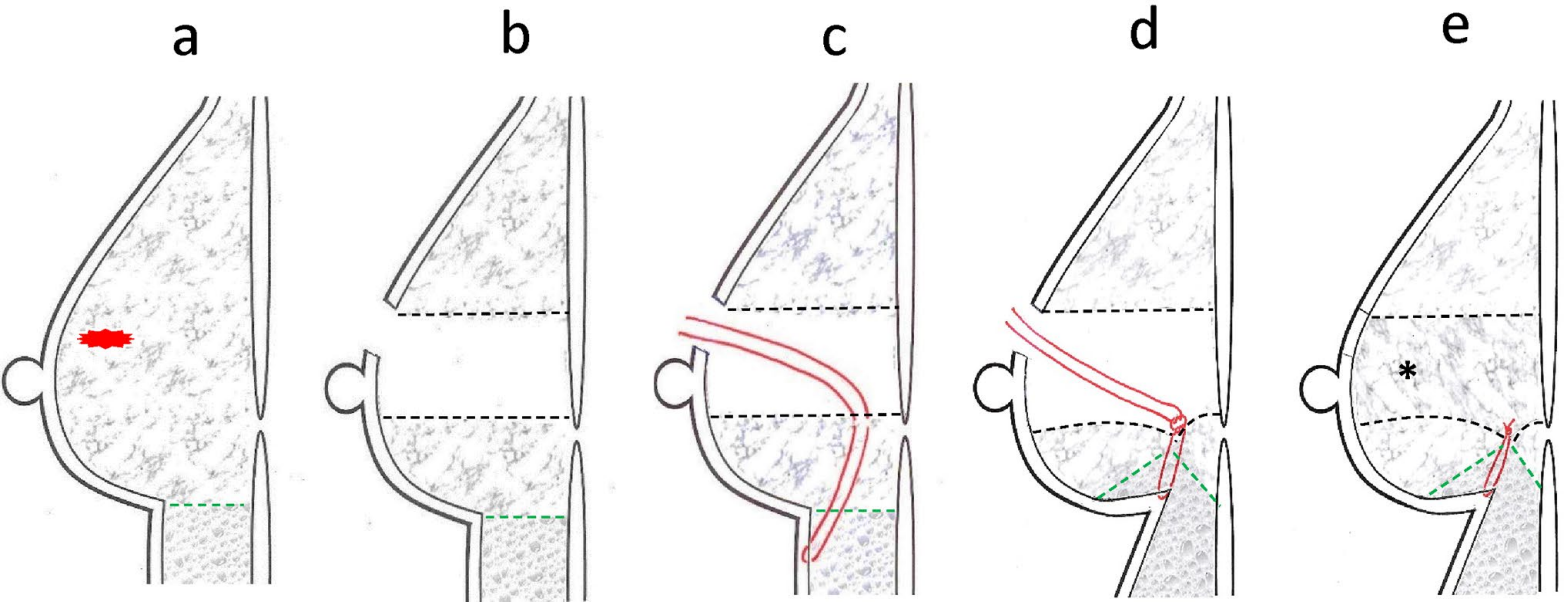
A schematic illustration of the surgical procedure from the vertical view. **a, b** A cylinder-shaped volume of breast tissue is removed with the overlying skin. **c** On the new inframammary line, stitches using 2–0 PDS^®^ were added to the subdermal layer. d. 2–0 PDS^®^ sutures were used to tie the breast tissue and subdermal fatty tissue together. e. The empty area is used to repair the skin-fatty-glandular tissue horizontally: Adjacent breast tissue and skin in Fig. 1. Dotted green line: the border between the breast tissue and inframammary fatty tissue. Dotted black line: the surgical edge of the remnant gland

A vertical view of the procedures is shown in Fig. 2.

### Adjuvant therapy

On a postoperative pathological examination, all margins were free from cancer. The patient received postoperative radiation therapy for the remnant glands as well as postoperative hormone therapy using an aromatase inhibitor, according to the guidelines for breast cancer treatment.

### Results

**Fig. 3:**
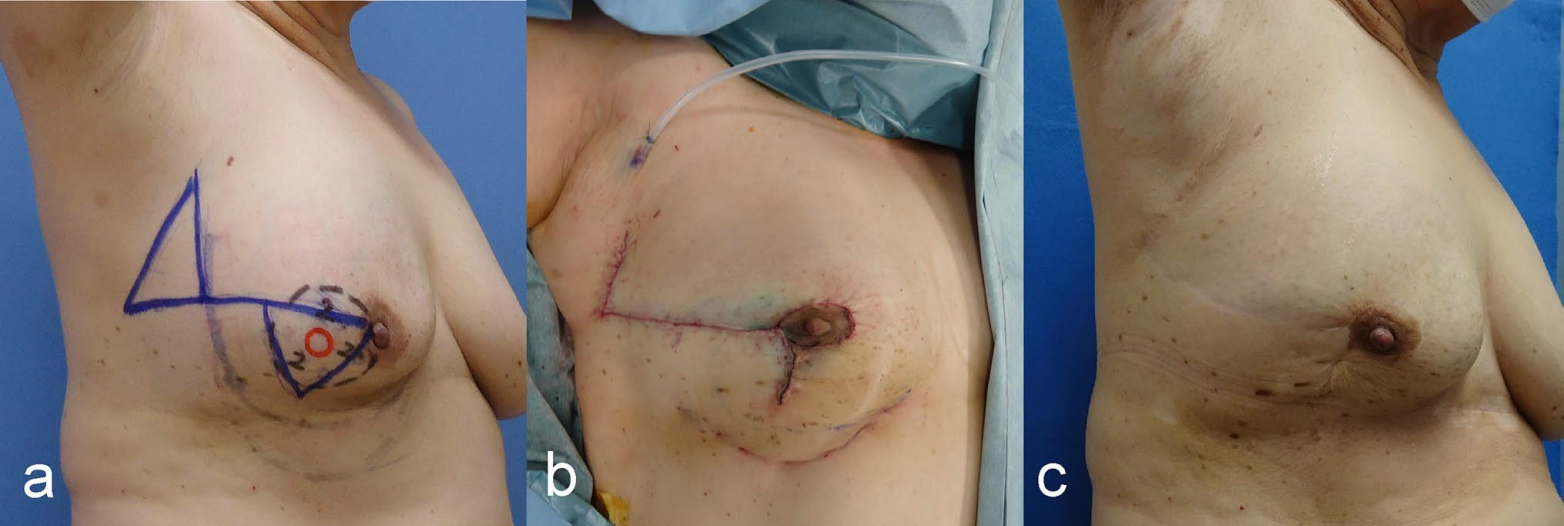
**a** Preoperative design, **b** postoperative findings, **c** 6 years after surgery

**Fig. 4:**
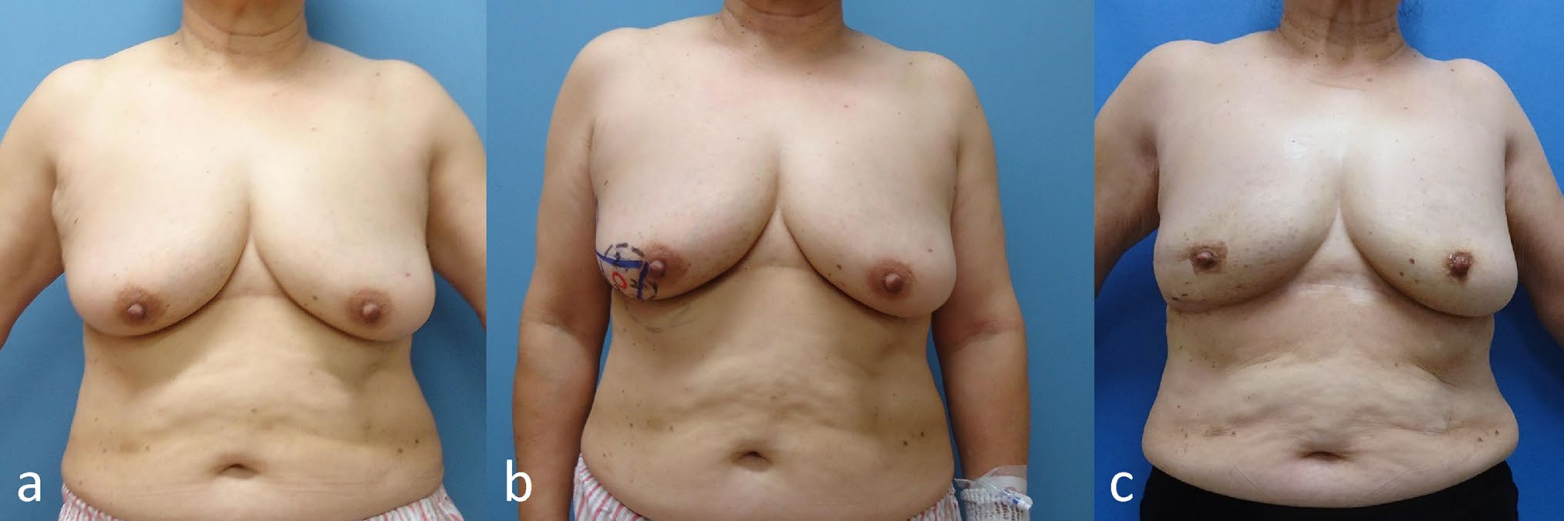
**a** Preoperative findings, **b** preoperative design, **c** 6 years after surgery

There were no postoperative complications such as bleeding, infection, fat necrosis, or blood flow disorder of the nipple-areola region. The observation period was 72 months without any local or distant recurrence. Good symmetry was obtained (Figs. 3, 4).

## Discussion

The OBS approach was established to achieve two major goals in the surgical treatment of breast cancer: oncological safety and excellent cosmetic results [2, 3]. With the introduction of OBS, we have achieved these goals in Japanese patients. For patients with small breasts that lack a sufficient volume for volume displacement, we developed techniques of volume replacement without using muscle [4–8]. We also revealed that OBS using the reduction mammoplasty technique was suitable for Japanese patients with large or ptotic breasts, as many researchers have reported in Western countries [9–11].

Some researchers have reported that the introduction of OBS to breast-conserving surgery resulted in improvements in not only the cosmetic result but also local control [12, 13]. When we remove the skin overlying the lesion due to cancer control for patients with a cancer lesion close to the nipple-areola, we select horizontal resection [14], a nipple-areolar grafting technique with inverted T-mammoplasty, or breast amputation [6, 15]. Excellent techniques have been developed to remove cancer lesions safely; however, they require the addition of contralateral breast surgery to obtain symmetry. We, therefore, sought a new technique to remove the cancer lesion with the over-lying skin completely, without the need for any contralateral operation.

With this technique, we removed an equilateral triangle of skin together with the cancerous and parenchymal breast tissue. There were two advantages to removing a cylinder-shaped volume of breast tissue along with the overlying skin: reducing the risk of remnant cancer lesions in the subdermal area, especially in cases of cancer lesions located in the superficial area of the breast, and avoiding undermining the wide area. If we remove only the breast tissue, an empty space appears under the conserved skin. This can be repaired by undermining a wide area or performing volume replacement [16]. However, if we remove the over-lying skin together, volume replacement or volume displacement using a skin-glandular flap can be safely performed, with excellent results achieved [17, 18]. In the present study, by horizontally sliding the skin-breast tissue toward the pyramidal space, we were able to avoid undermining the breast tissue for volume displacement. The nipple was, therefore, not pulled in the direction where the breast volume was lost. Of note, for patients with large breasts and a slim body, some discrepancy might appear at the lateral edge of the Z-scar due to the difference in the thickness of the breast and the lateral thoracic fatty tissue.

We added several sutures to make the inframammary line clear to help maintain the breast shape. As shown in Fig. 2, our procedure is a modified version of the technique reported by Ogawa et al. Those authors reported that the combination of the creation of a neoinframammary line and the extended mobilization of the gland flaps helped create breast volume [16]. We modified this technique not for volume creation but to maintain symmetry of the bilateral inframammary line level. In this manner, we were able to maintain a natural shape of the breast, and this shape was retained even without fixation to the chest wall using the interrupted sutures inserted into the dermis layer on the new inframammary line in our case. We routinely use this technique at the time of volume replacement and have achieved excellent results. However, we have not yet reported our experience to reveal how effectively this technique provides good cosmetic results in OBS. We are aware of the importance of reporting such data and plan to publish our findings in the near future.

The other negative aspect of this technique is the relatively conspicuous scar left on the lateral area of the breast and axilla. When we determined the indication of this OBS, we consider our patient’s opinion concerning scar formation. However, the incision scar was actually less conspicuous than expected. In the standing position, with the arm down, half of the Z-scar is inconspicuous from the frontal view as well as from the viewpoint of the patient.

Given the abovementioned advantages and disadvantages, we feel that this technique is adequate for patients with fatty breasts, a cancer lesion in the central or lateral area, and an oncological reason for removing the overlying skin. Furthermore, this technique is suitable for patients who do not wish to receive volume replacement using a musculo-cutaneous flap or volume displacement with contralateral healthy breast surgery.

## Conclusion

A new technique of OBS combining partial mastectomy with resection of double equilateral triangular skin flaps was safely performed, and the cosmetic result was excellent.
